# *Bufonis venenum* extract loaded novel cholesterol-free liposome for the treatment of hepatocellular carcinoma

**DOI:** 10.3389/fphar.2024.1486742

**Published:** 2024-11-25

**Authors:** Siqi Yang, Jinshuai Lan, Zhe Li, Ming Li, Ya Wu, Liyan Sun, Tong Zhang, Yue Ding

**Affiliations:** ^1^ School of Pharmacy, Shanghai University of Traditional Chinese Medicine, Shanghai, China; ^2^ State Key Laboratory of Integration and Innovation of Classic Formula and Modern Chinese Medicine, Shanghai University of Traditional Chinese Medicine, Shanghai, China; ^3^ National Innovation Platform for medical industry-education integration, Shanghai University of Traditional Chinese Medicine, Shanghai, China

**Keywords:** hepatocellular carcinoma, *Bufonis venenum*, liposomes, cell apoptosis, traditional Chinese medicine

## Abstract

**Background:**

This study aims to improve the solubility and the toxicity of Bufonis venenum, and finally enhance the therapeutic outcomes of hepatocellular carcinoma (HCC).

**Methods:**

The cholesterol-free liposomes simultaneously encapsulate bufadienolides and indolealkylamines (Non-Cholesterol-Bufonis Venenum Extract-Liposome, Non-Chol-BVE-LP) was prepared by the thin-film evaporation technique. *In vitro*, the cytotoxicity, cell apoptosis study, cellular uptake and hemolysis studies were evaluated in HepG2 cells. *In vivo*, the biodistribution and anti-tumor activity studies were conducted in BALB/C mice with HepG2 cells.

**Results:**

The liposomes showed good size distribution, encapsulation efficiency drug loading capacity and slower drug release. Non-Chol-BVE-LP had higher cytotoxicity on HepG2 cells and induced more apoptosis on HepG2 Cells compared with BVE. In addition, the liposomes could accumulate in tumor by passive targeting, thus facilitating the anti-tumor effects. *In vivo*, Non-Chol-BVE-LP showed equivalent anti-tumor efficacy to the first-line anti-HCC drug sorafenib.

**Conclusion:**

The study provided new ideas for the development and clinical application of Bufonis venenum related formulation and offered new drug for the treatment of HCC.

## 1 Introduction

Hepatocellular carcinoma (HCC) is the sixth most commonly diagnosed cancer with mortality rate ranking the third among all cancer (Sung et al., 2021; Xie et al., 2023). HCC is susceptible to intrahepatic and extrahepatic metastases. This makes the clinical prognosis of patients worse (Jia et al., 2023). Currently, the main diagnostic methods of HCC are ultrasounds, enhanced CT or MRI, tissue biopsy, and identification of biomarkers (Zhang et al., 2022). The treatment of HCC are modalities urgical resection, liver transplantation, local ablation, hepatic artery chemoembolization (TACE) and systemic therapy (Ajoolabady et al., 2023; Forner et al., 2018; Vogel et al., 2019; Vogel and Saborowski, 2022). Since most patients are diagnosed with advanced HCC and not suitable for surgical treatment, systemic therapy is usually the only chance for them. However, there are few options for systemic therapy for advanced HCC with only two first-line drug, Sorafenib and Lenvatinib, available. But both Sorafenib and Lenvatinib have poor effects on improving the survival rate of HCC patients, and it has been found that many HCC patients show resistance to these drug (Lu et al., 2022; Tang W. et al., 2020). Consequently, the development of innovative therapeutic modalities is imperative to enhance the therapeutic outcomes for patients diagnosed with HCC. Modern pharmacological researches show that some traditional Chinese medicine can act in all aspects of tumor occurrence and development, and has unique advantages in the treatment of tumors (Chen et al., 2022; Ge et al., 2018; Li et al., 2018; Luo et al., 2020; Tang K. Y. et al., 2020).

**FIGURE 1:**
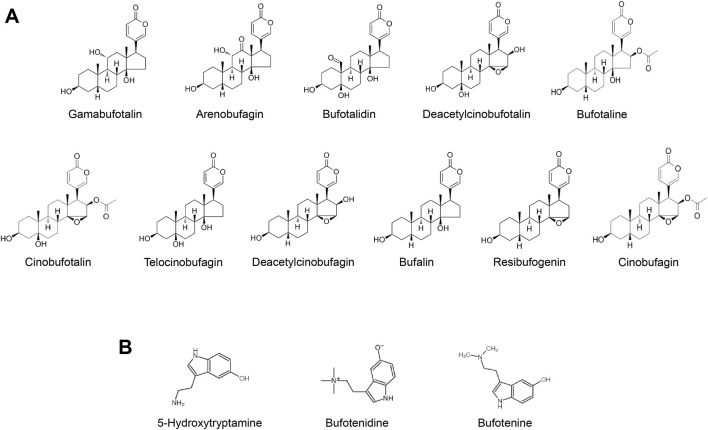
**(A)** Typical bufadienolides in *Bufonis venenum* **(B)** Typical indolealkylamines in *Bufonis venenum*.

*Bufonis venenum*, which is the dried secretion of *Bufo bufo gargarizans Cantor* or *Bufo melanostictus*, has been widely used in the treatment of malignant tumors, including hepatocellular carcinoma, lung carcinoma, gastric carcinoma and other malignant tumors (Nalbantsoy et al., 2016; Wei et al., 2019; Zhu et al., 2018). The extract of *Bufonis venenum* mainly contains bufadienolides and indolealkylamines (Bi et al., 2016; Tao et al., 2023) (Figures 1A, B). In recent years, studies have shown that bufadienolides has certain anti-tumor effects. It can promote the apoptosis of tumor cells and reverse the drug resistance of tumor cells (Dai et al., 2023; Yun et al., 2009; Zhang et al., 2012). It has been reported that Cinobufagin and Bufalin can induce apoptosis via Fas- and mitochondria-mediated pathways (Qi et al., 2011). Gamabufotalin can improves the suppressive tumour immune microenvironment by inhibiting PD-L1 (Fan et al., 2024). Angiogenesis is proven to be an inhibitor of VEGF-mediated angiogenesis (Li et al., 2012). Resibufogenin can inducing reactive oxygen species accumulation and thus kill tumour cells (Han et al., 2018). Indolealkylamines has anti-inflammatory and immune-boosting effects (Xu et al., 2021; Zhang et al., 2019). It has been reported that 5-hydroxytryptamine can promote liver regeneration and immune cell activation (Fang et al., 2018; Karmakar and Lal, 2021). Bufotenine and bufotenidine have analgesic activilities (Xu et al., 2021). Both the Huachansu capsules (which mainly contain bufadienolides) and Huachansu injection (which primarily contains indolealkylamines) have exhibited significant anti-HCC activities in clinic. However, bufadienolides has poor solubility in water and tends to be rapidly distributed and eliminated *in vivo*. Furthermore, it may induce adverse reactions such as accelerated heart rate, convulsions, nausea and vomiting. Since the hydrophobicity limits the clinical use of bufadienolides to some extent. It is of great importance to improve the bioavailability and reduce the toxicity of these compounds through advancements in pharmaceutical technology.

Nanotechnology has demonstrated significant ability to delivery insoluble drug *in vivo* and reduce the toxicity of drug (Farokhzad and Langer, 2009). Liposomes, as a kind of drug carriers, have been widely used in clinic. They have biofilm-like properties with good tissue permeability and targeting. Besides, they can encapsulate lipophilic drug in the phospholipid bilayer and hydrophilic drug in the hydrophilic core, which can protect the encapsulated macromolecules from degradation, play a role in reducing the toxicity and improve the therapeutic efficacy of the drug (Abu Lila and Ishida, 2017; Mazur et al., 2017; Wang et al., 2009). Liposomes are generally composed of phospholipids and cholesterol (Maeda et al., 2017). Cholesterol can regulate the fluidity of the phospholipid bilayer membrane and retard the lateral flow of oxidized phospholipids (Kaddah et al., 2018). The above mechanism prevents the deformation of liposomes and the creation of air pockets, which can keep a relatively robust bilayer structure of liposomes even after a certain degree of oxidation. However, taking too much cholesterol may cause cardiovascular disease, atherosclerosis and other health problems (Folkerd and Dowsett, 2010; Nelson et al., 2014; Wu et al., 2019). Steroids and saponins, which have similar structure to cholesterol, are capable of replacing cholesterol at right proportions without damaging the structure of liposomes (Hong et al., 2019; Li et al., 2020; Lu et al., 2018). The bufadienolides in *Bufonis venenum* extract is such a kind of steroid.

In this study, we aim to prepare the cholesterol-free liposomes by replacing cholesterol with bufadienolides, which has a similar chemical structure to cholesterol, and co-encapsulate indolealkylamines in the hydrophilic core of the liposomes. The characterization, uptake ability, *in vitro* and *in vivo* anti-tumor effects and *in vivo* biodistribution of the liposome was investigated, This study is expected to provide new ideas for the development and clinical application of *Bufonis venenum* related formulation and to offer new drug for the treatment of HCC.

## 2 Materials and methods

### 2.1 Reagents

DSPC, DSPE-mPEG2000 and cholesterol were obtained from AVT (Shanghai) Pharmaceutical Tech Co., Ltd., (Shanghai, China). *Bufonis Venenum* Extract was extracted and isolated according to an established procedure. Chloroform, methanol, rhodamine B (RB), indocyanine green (ICG) and Phosphotungstic acid were obtained from Sinopharm Chemical Reagent Co., Ltd., (Shanghai, China). Sephadex G-50 Dextran Gel was obtained from Pharmacia LKB Biotechnology Inc., (Uppsala, Sweden). MEM medium, FBS and Penicillin-Streptomycin solution were obtained from Thermo Fisher Scientific (Waltham, United States of America). Cell Counting Kit-8 (CCK-8) was obtained from Shanghai Biyuntian Biological Co., Ltd., (Shanghai, China). Gamabufotalin, Arenobufagin, Bufotalidin, Deacetylcinobufotalin, Bufotaline, Cinobufotalin, Telocinobufagin, Deacetylcinobufagin, Bufalin, Resibufogenin, Cinobufagin and 5-Hydroxytryptamine were obtained from Shanghai Hongyong Biological Co., Ltd., (Shanghai, China). Bufotenidine and Bufotenine were obtained from Anhui Xiqingguo Biological Co., Ltd., (Anhui, China).

### 2.2 Cells

HepG2 cells were obtained from the Cell Bank of Typical Culture Preservation Committee of the Chinese Academy of Sciences (Shanghai, China) and cultivated in complete MEM medium (containing 10% FBS and 1% Penicillin-Streptomycin solution). The cells were incubated at 37°C in 5% CO_2_ incubator for subsequent experiments.

### 2.3 Animals

Male Sprague Dawley rats were obtained from Shanghai Sippr bk laboratory animal Co., Ltd., (Shanghai, China). Male BALB/C mice were obtained from SLAC Laboratory Animal Co., Ltd., (Shanghai, China). All institutional and national guidelines for the care and use of laboratory animals were followed.

### 2.4 UPLC and HPLC conditions

Agilent 1290 series UPLC with a column (Agilent Eclipse Plus C18 RRHD, 100 mm × 2.1 mm, 1.8 μm) and an ultraviolet detector (300 nm) was used to quantify the bufadienolides concentration. The column temperature was maintained at 35°C. The separation system was consisted of 0.1% phosphoric acid in water (A) and methanol (B). The solvent gradient was set as follows: 0–5 min, 15% B; 5–10 min, 15%–18% B; 10–15 min, 18% B; 15–17 min, 18%–40% B; 17–25 min, 40%–55% B; 25–28 min, 55%–75% B; 28–30 min, 75%–90% B and 30–35 min, 90% B. The injection volume was 2 μL, and the flow rate was approximately 0.4 mL/min.

Agilent 1200 series HPLC with a column (Diamonsil Plus C18, 250 mm × 4.6 mm, 5 μm) and an ultraviolet detector (275 nm) was used to quantify the indolealkylamines concentration. The column temperature was maintained at 30°C. The separation system was consisted of 0.5% potassium dihydrogen phosphate in water (A) and methanol (B). The solvent gradient was set as follows: 0–5 min, 8% B; 5–20 min, 8%–12% B; 20–25 min, 12%–80% B and 25–35 min, 80% B. The injection volume was 10 μL, and the flow rate was approximately 1 mL/min.

### 2.5 Preparation of liposomes

*Bufonis Venenum* Extract (BVE) was extracted and isolated according to an established procedure (Lee et al., 2012). We have improved on this method. Bufonis venenum was added to 70% ethanol (ratio 1:120) and refluxed for 140 min 2 times. After filtration and evaporation, the final BVE was obtained. The content of main components of extract was test by HPLC and supplied in SI (Supplementary Table S1).

Non-Cholesterol-BVE-Liposome (Non-Chol-BVE-LP) was prepared by the thin-film evaporation technique. After a preliminary prescribing study (Supplementary Figures S1, S2), Non-Chol-BVE-LP was consisted of 28.16 mg of DSPC, 25 mg of DSPE-mPEG2000 and 4.28 mg of Bufonis Venenum extract (BVE). The above mixture was dissolved respectively in chloroform/methanol (1:1, v/v) and evaporated to get a thin lipid film under 60°C by rotatory evaporator. To ensure organic reagents were completely removed, the lipid film was kept in a vacuum dryer overnight. 4 mL of phosphate buffered saline (PBS, pH 6.8) was added to hydrate the lipid film. The above hydrated mixture solution was probe-sonicated in an ice-water for 10 min (80 W, 3S on/3S off) to get Non-Chol-BVE-LP.

Rhodamine B (RB) and Indocyanine Green (ICG) liposomes were prescribed by loading RB or ICG into the Non-Chol-LP. The rest of the steps were the same as the preparation of Non-Chol-BVE-LP.

### 2.6 Characterization of liposomes

The particle size and zeta potential of the liposomes were characterized using a Zetasizer Nano ZS (Malvern Instruments Ltd, United Kingdom). 50 μL of liposome suspension was diluted with 1 mL of double-distilled water before measuring. The morphology of the liposomes was visualized under a transmission electron microscope (TEM). The encapsulation efficiency (EE) and drug loading (DL) of the liposomes were determined by dextran gel G-50 column chromatography. The concentration of components in Non-Chol-BVE-LPs was determined by ultraperformance liquid chromatography (UPLC) at 300 nm. The following equations were used for the calculations:

$$\text{EE }\left(\%\right)=\left(\text{drug encapsulated in liposomes}/\text{total drug for the preparation of liposomes}\right)\times 100\%$$

$$\text{DL }\left(\%\right)=\left(\text{drug encapsulated in liposomes}/\text{total weight of liposomes}\right)\times 100\%$$

All measurements were performed in triplicate respectively.

### 2.7 Stability of liposomes

The stability of liposomes was investigated by particle size and EE. The liposomes prepared were stored in dark at 4°C. Samples were collected at 1, 7, 14, 21 and 30 d to determine the above indicators.

### 2.8 *In vitro* drug release

The *in vitro* drug release of liposomes was examined by dialysis method. 2 mL of Non-Chol-BVE-LP and BVE solution were added into a dialysis bag (molecular weight: 7 KDa) and sealed respectively. The dialysis bags were immersed into 200 mL of PBS with 0.5% Tween-80 and placed on a magnetic stirrer (37°C, 100 rpm). After measuring the volume of liposomes in the dialysis bags, 0.1 mL of the solution in the bag was taken to determine the drug concentration by UPLC. Samples were collected at the time point of 10, 20, 30, 45, 60, 120, 240, 360 and 480 min. The drug release profiles of preparation and free drug were plotted and compared by calculating the cumulative percentage of drug release ratio at each sampling time.

### 2.9 Cytotoxicity

The cytotoxicity of Non-Chol-BVE-LP and BVE was evaluated by CCK8 assay. 100 μL of HepG2 cell or LO2 cell suspension (1 × 10^5^ cells/mL) was inoculated into 96-well plates and incubated at 37°C in 5% CO_2_ incubator overnight. Different concentrations of BVE solution and Non-Chol-BVE-LP were added into 96-well plates in three replicates. After 48 h of incubation, 10 μL of CCK-8 solution was added into per well. After incubating for another 2 h in the 5% CO_2_ incubator, the optical density (OD) of each well was measured at 450 nm by a microplate reader. The cell inhibition rate was calculated as following equation:

$$\text{Cell }{\text{inhibition}\;\left(\%\right)=[1-(\text{OD}}_{\text{treatment}}{-\text{OD}}_{\text{blank}}{)\;/\;(\text{OD}}_{\text{control}}{-\text{OD}}_{\text{blank}})]\times 100\%$$

### 2.10 Cell apoptosis study

Flow cytometer was used to detect the apoptosis of HepG2 cells. 2 mL of HepG2 cell suspension (3 × 10^5^ cells/mL) was inoculated into 6-well plates and incubated at 37°C in 5% CO_2_ incubator overnight. After discarding the culture medium, 2 mL of BVE solution or Non-Chol-BVE-LP was added into each well. The concentration of BVE and Non-Chol-BVE-LP were 0.18 μg/mL and 0.10 μg/mL. Cells were incubated for 48 h and then washed with 2 mL of PBS (containing 1% FBS). Cells in each well were digested with 200 μL of trypsin (without EDTA) and resuspended into 100 μL of Binding Buffer. 5 μL of Annexin V-FITC was added into the cell solutions followed by incubating in dark at 4°C for 30 min 5 min before testing by flow cytometry, 400 μL of Binding Buffer and 5 μL of PI were added.

### 2.11 *In vitro* cellular uptake studies

The flow cytometry and confocal laser scanning microscope (CLSM) were employed to analyze the cellular uptake of preparations by HepG2 cells. For flow cytometry study, HepG2 cell suspension (1 × 10^5^ cells/mL) was inoculated into 6-well plates in three replicates and incubated at 37°C in 5% CO_2_ incubator overnight. After discarding the culture medium, 2 mL of culture medium with RB or Non-Chol-RB-LP solution was added. The concentration of RB and BVE was 5 μg/mL and 0.5 μg/mL. After 2 h of incubation, the culture medium was discarded, and cells were washed three times with PBS. Cells in each well were digested with trypsin and resuspended into 1 mL of PBS. The cell suspensions were transferred into a flow tube for detection.

For CLSM study, HepG2 cell suspension (1 × 10^5^ cells/mL) was inoculated into glass-bottom Petri dishes in three replicates and incubated at 37°C in 5% CO_2_ incubator overnight. After discarding the culture medium, 2 mL of culture medium with RB or Non-Chol-RB-LP solution was added. The concentration of RB and BVE was 5 μg/mL and 0.5 μg/mL. After 2 h of incubation, the culture medium was discarded, and cells were washed three times with PBS. 1 mL of 4% paraformaldehyde solution was used to fix the cells for 10 min. After washing three times with PBS, 500 μL of Hoechst 33342 was used to perform nuclear staining for 20 min. The fluorescent dyes were discarded, washed three times with PBS and 500 μL of PBS was added to each dish. Finally, the cells were observed and photographed with a CLSM.

### 2.12 *In vitro* hemolysis study

The erythrocytes of SD rats were collected. Pure water (positive control), PBS solution (negative control), BVE solution, and Non-Chol-BVE-LP was added respectively to centrifuge tubes containing 20 μL of erythrocytes. After mixing, the mixtures were centrifuged at 3,500 rpm for 10 min to get the supematant after keeping at room temperature for 30 min. A UV-visible spectrophotometry was used to measure the absorbance of the supematant at a wavelength of 540 nm and the hemolysis rates were calculated.

### 2.13 *In vivo* biodistribution and anti-tumor activity of liposomes

A HepG2 subcutaneous tumor mouse model of hepatocellular carcinoma was established to investigate the biodistribution and anti-tumor activity of the preparations. Healthy male BALB/C mice with a body weight of approximately 20 ± 2 g were kept in the laboratory for 7 days to adapt before the study. HepG2 cells were digested with trypsin and resuspended into serum-free medium (3 × 10^6^ cells/mL). 125 μL of HepG2 cell suspension were subcutaneously injected into the right axilla of each BALB/c mouse.

The biodistribution study was conducted on tumor-bearing BALB/c mice. When the tumor volume reached about 100 mm^3^, the mice were randomly divided into free ICG group and Non-Chol-ICG-LP group. Free ICG or Non-Chol-ICG-LP were intravenously administrated at an equal ICG dose of 2 mg/kg and BVE dose of 0.08 mg/kg, followed by anesthetizing the mice with 1.5% isoflurane, and the fluorescence distribution and intensity of ICG in the mice was recorded at 1, 2, 4, 6, 8, and 24 h post injection using the IVIS Lumina *in vivo* imaging system. After 24 h of the administration, the mice were sacrificed. Tumors, hearts, livers, spleens, lungs, and kidneys were removed and flushed by saline for *ex vivo* fluorescence imaging.

The *in vivo* anti-tumor study was conducted on tumor-bearing BALB/c mice. When the tumor volume reached about 50 mm^3^, the mice were randomly divided into the following five groups: control (saline), sorafenib, Chansu injection, BVE and Non-Chol-BVE-LP. The dose of sorafenib, Chansu injection and BVE were 30 mg/kg, 20 μL/g and 0.08 mg/kg. All groups were treated every other day. The body weights and tumor volumes of the mice were recorded every other day. The tumor volume was calculated as the following equation:

$$\text{Tum}{\text{or}\;\text{volume}\;(\text{mm}}^{3})=\pi \;/\;6\times {\;\text{width}}^{2}\times \text{length}$$

After 2 weeks of the treatment, the mice were sacrificed 24 h after the last treatment. The tumors and hearts were excised. After weighing the tumor, the tumor inhibition rate was calculated by the following equation:

$$\text{Tumor inhibition }\left(\%\right)=\left[\left({\text{Tumor}\;\text{weight}}_{\text{control}}-{\text{Tumor}\;\text{weight}}_{\text{treatment}}\right){\;/\;\text{Tumor}\;\text{weight}}_{\text{control}}\right]\times 100\%$$

All the tissues were flushed by saline and stored at 4% paraformaldehyde fix solution for 48 h. Samples were fixed in paraffin and sliced into 3–5 µm sections then. After dehydrating overnight, hematoxylin and eosin staining (H&E) was conducted. Then perform histological observations with light microscope.

### 2.14 Statistical analysis

Data were expressed as mean value ± standard deviation (SD). Differences among groups were performed with a one-way analysis of variance (ANOVA) test. *P* < 0.05 was considered statistically significant.

## 3 Results

### 3.1 Characterization of *Bufonis venenum* extract loaded liposomes

**FIGURE 2:**
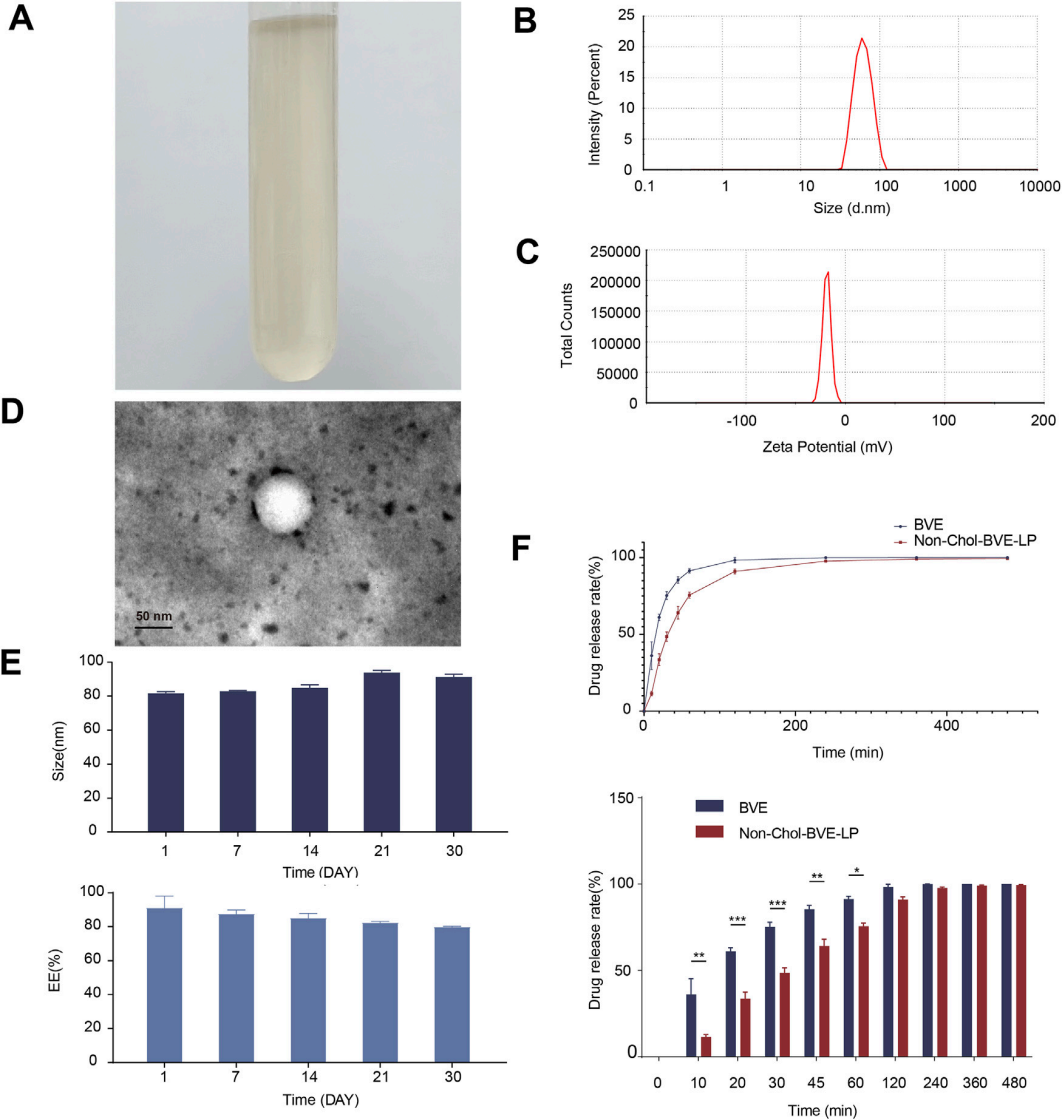
**(A)** The appearance of Non-Chol-BVE-LP **(B)** Particle size of Non-Chol-BVE-LP **(C)** Zeta potential of Non-Chol-BVE-LP **(D)** TEM images of Non-Chol-BVE-LP **(E)** Changes in particle size and EE of Non-Chol-BVE-LP during 30 days (n = 3). **(F)** *In vitro* drug release of BVE and Non-Chol-BVE-LP (n = 3).

The appearance of Non-Chol-BVE-LP was a slightly yellowish clarified solution (Figure 2A). Non-Chol-BVE-LP was prepared with the particle size of 81.51 ± 1.10 nm and the zeta potential of −20.1 ± 0.76 (Figures 2B, C). The concentration of Gamabufotalin, Arenobufagin, Bufotalidin, Deacetylcinobufotalin, Bufotaline, Cinobufotalin, Telocinobufagin, Deacetylcinobufagin, Bufalin, Resibufogenin, Cinobufagin, 5-Hydroxytryptamine, Bufotenidine, Bufotenine in Non-Chol-BVE-LP were 11.12 ± 0.10 μg/mL, 18.22 ± 0.43 μg/mL, 6.25 ± 1.85 μg/mL, 0.45 ± 0.03 μg/mL, 18.76 ± 1.30 μg/mL, 20.82 ± 0.86 μg/mL, 8.94 ± 0.77 μg/mL, 1.99 ± 0.09 μg/mL, 29.83 ± 2.40 μg/mL, 40.25 ± 0.80 μg/mL, 56.40 ± 3.44 μg/mL, 0.62 ± 0.21 μg/mL, 3.89 ± 0.97 μg/mL and 0.09 ± 0.04 μg/mL respectively.

The EE of Non-Chol-LP was 91.20% ± 6.80% and the DL was 2.87% ± 0.11%. TEM was used to evaluate the appearance and shape of the liposomes. The micrograph showed that the Non-Chol-BVE-LP was spherical with relatively uniform size. And no aggregation or fusion was observed (Figure 2D). The stability of the Non-Chol-BVE-LP in PBS during 30 days was investigated by particle size and encapsulation efficiency (Figure 2E). The stability of the Non-Chol-BVE-LP in saline and DMEM during 7 days was investigated either (Supplementary Figure S3). The particle size of Non-Chol-BVE-LP in saline was 76.66 ± 0.27 nm. The particle size of Non-Chol-BVE-LP in MEM was 80.27 ± 0.42 nm. The EE of Non-Chol-BVE-LP in saline was 93.59% ± 1.91%. The particle size of Non-Chol-BVE-LP in MEM was 93.66% ± 3.38%. The particle size and EE of the liposomes in saline and MEM during 7 days were stable. In general, the stability of Non-Chol-BVE-LP was good. There was no significant difference between 1 d and 30 d in particle size or encapsulation efficiency, which indicated that liposomes did not aggregate or degrade under a certain time of storage.

### 3.2 *In vitro* drug release

The *in vitro* release profiles of free drug and liposomes over 480 min were shown (Figure 2F). After 30 min, 75.3% ± 2.6% of free drug was released, while in Non-Chol-BVE-LP, only 48.5% ± 3.1% of the drug was released. In 60 min, 75.6% ± 1.9% of the drug was released from Non-Chol-BVE-LP. These results indicated that liposome had a certain sustained-release effect on the encapsulated drug, which could lower the C_max_ of the drug in blood after injection and reduce the toxicity caused by high blood concentration of *Bufonis venenum* extract.

### 3.3 Cytotoxicity and cell apoptosis study

**FIGURE 3:**
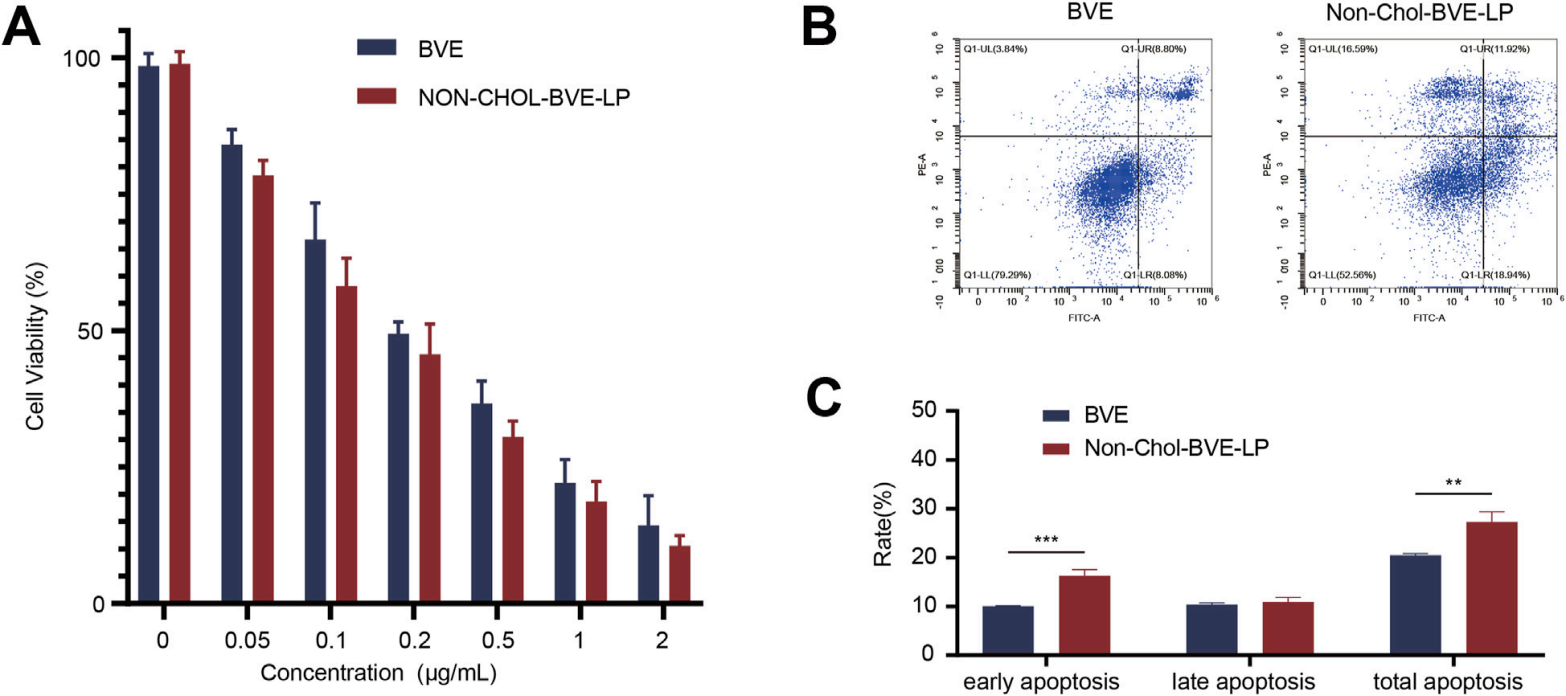
**(A)** Cytotoxicity of BVE and Non-Chol-BVE-LP on HepG2 cells **(B)** Flow cytometry analysis of apoptosis on HepG2 cells **(C)** The quantitative analysis results according to the flow cytometry analysis (n = 3, *P < 0.05, **P < 0.01, ***P < 0.001).

*Bufonis venenum* extract has been reported to be able to kill various tumor cells and induce apoptosis in tumor cells, which is also its main mechanism of anti-tumor activity. *In vitro* cytotoxicity of BVE and Non-Chol-BVE-LP on HepG2 cells was measured by CCK8 assay (Figure 3A). After 48 h of incubation, the cell viability of HepG2 cells was negatively correlated with the concentration of the drug. According to the results of cytotoxicity, IC_50_ values of BVE solution and Non-Chol-BVE-LP were 0.24 μg/mL and 0.17 μg/mL respectively. Compared with BVE solution, Non-Chol-BVE-LP group showed higher cytotoxicity on HepG2 cells. *In vitro* cytotoxicity of BVE and Non-Chol-BVE-LP on LO2 cells was measured by CCK8 assay (Supplementary Figure S4). The result indicated that BVE and Non-Chol-BVE-LP does not cause significant toxicity to normal liver cells even at four times the therapeutic doses on HepG2 cells. Annexin V-FITC/PI double staining was used to detect apoptosis on HepG2 cells (Figures 3B, C). For BVE group and Non-Chol-BVE-LP group, the total apoptosis rates were 20.46% ± 0.35% and 27.26% ± 2.10% respectively. The early apoptosis rate, late apoptosis rate and total apoptosis rates of BVE group were lower than Non-Chol-BVE-LP, which was consistent with the results of cytotoxicity.

### 3.4 *In vitro* cellular uptake studies

**FIGURE 4:**
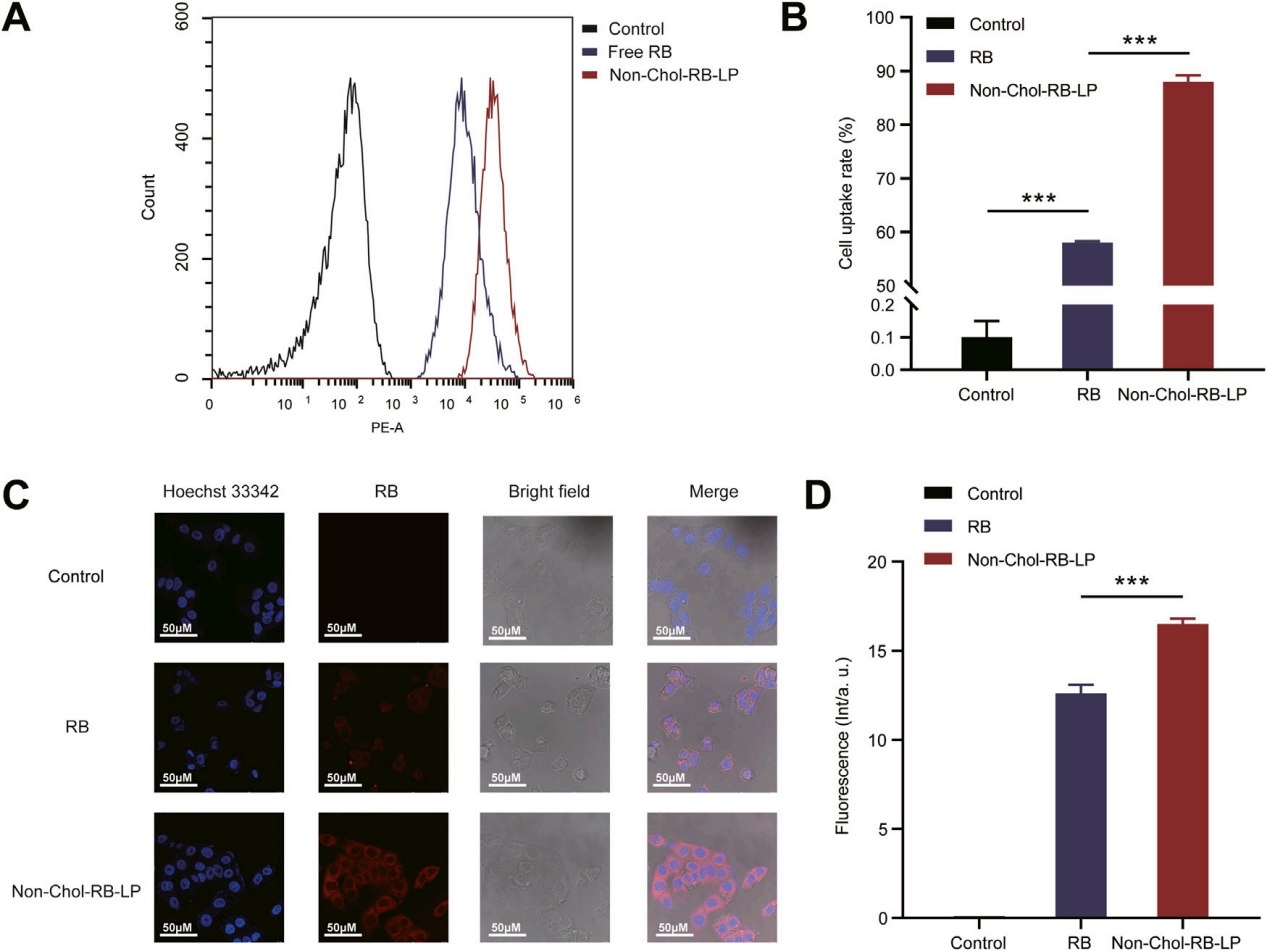
**(A)** *In vitro* cellular uptake of BVE and Non-Chol-BVE-LP by HepG2 cells **(B)** The quantitative analysis results according to the uptake **(C)** CLSM images of cells incubated with RB and Non-Chol-BVE-LP **(D)** The quantitative analysis results according to the CLSM analysis Data is expressed as mean ± SD (n = 3, *P < 0.05, **P < 0.01, ***P < 0.001).

RB was used to mark the liposome for cellular uptake studies. The analysis results of flow cytometry were shown (Figures 4A, B). The uptake of Non-Chol-RB-LP by HepG2 cells was significantly higher than that of RB. The results were further verified by CLSM (Figures 4C, D). The red fluorescence was the staining of RB and blue fluorescence was the nucleus after being stained by Hoechst 33342. The increased intracellular red fluorescence indicated the increased uptake of RB by HepG2 cells. The uptake of Non-Chol-RB-LP by HepG2 cells was also higher than that of RB, which was in agreement with those obtained by flow cytometry.

### 3.5 *In vivo* biodistribution and anti-tumor activity of liposomes

**FIGURE 5:**
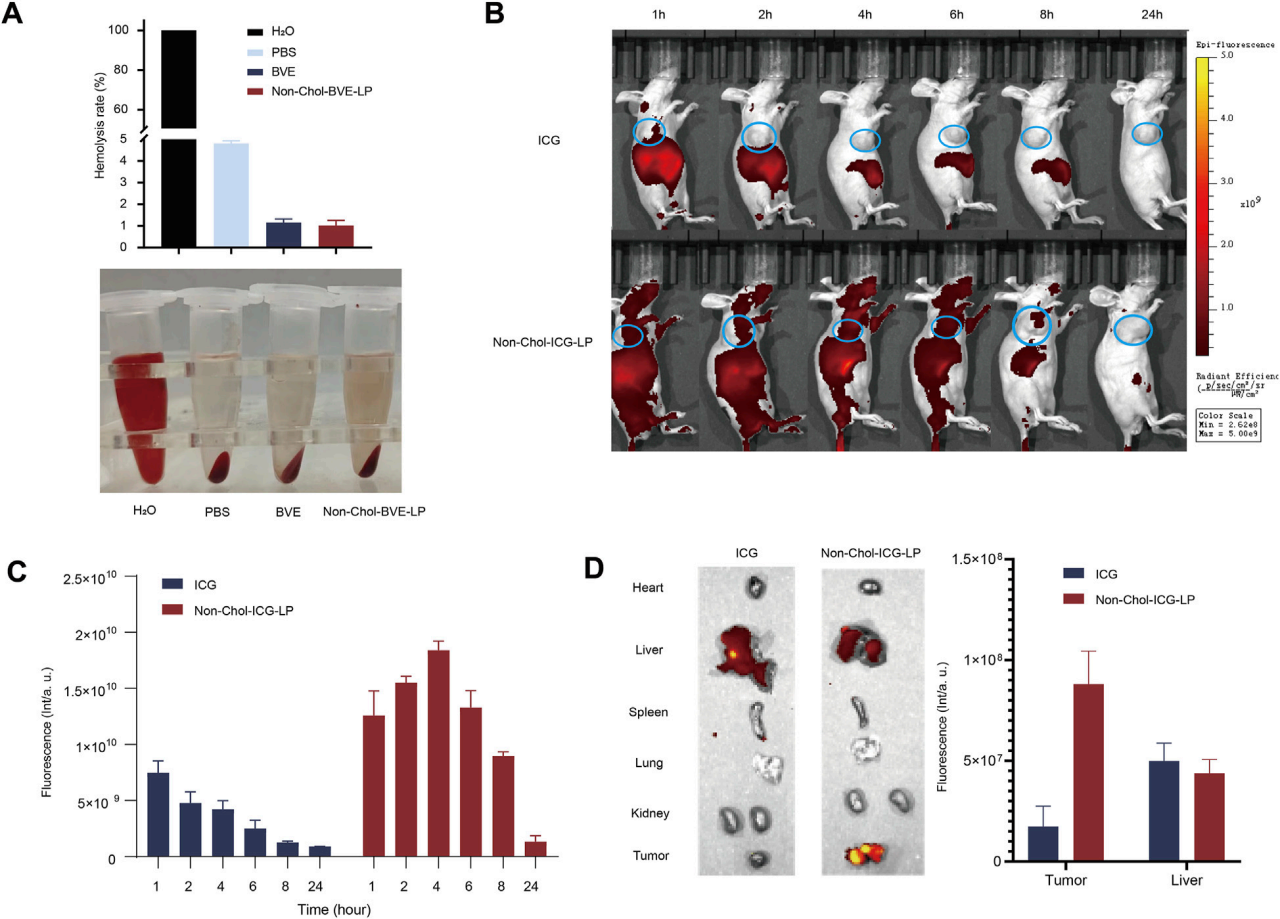
**(A)** Hemolysis in each group **(B)** *In vivo* biodistribution images of ICG and Non-Chol-BVE-LP **(C)** The fluorescence semi-quantification analysis results of tumor during 24 h after the administration **(D)** *Ex vivo* fluorescence images of organs and tumors at 24 h after administration and the fluorescence semi-quantification analysis results according to the biodistribution analysis. Data is expressed as mean ± SD (n = 3, *P < 0.05, **P < 0.01, ***P < 0.001).

Before *in vivo* studies, we first investigated the hemolytic activity of the formulations. As it was shown (Figure 5A), the hemolysis rates of BVE and Non-Chol-BVE-LP were less than 5% at the actual *in vivo* dose, meeting the requirements for intravenous injection. BVE and Non-Chol-BVE-LP demonstrated good blood safety when administered *in vivo*. ICG was used to mark the liposome for *in vivo* distribution studies. The *in vivo* distribution of free ICG and Non-Chol-ICG-LP in nude mice was examined by an IVIS Lumina *in vivo* imaging system. Equal doses of ICG solution and Non-Chol-ICG-LP were injected via tail vein. As it was shown (Figures 5B, C), the fluorescence intensity was positively correlated with time within 4 h after administration, and then declined. Comparing with ICG group, Non-Chol-ICG-LP was distributed more widely *in vivo* 4 h after administration, which indicated liposomes could prolong the *in vivo* circulation of drug. In isolated tumors of the mice (Figure 5D), the fluorescence intensity of ICG group was weaker than that of Non-Chol-ICG-LP group. And in isolated organs, fluorescence intensity was almost absent except in livers. Because the liver, which was rich in reticuloendothelial cells distributed throughout, was capable of ingesting liposomes and was also the primary organ for drug metabolism. These results indicated that Non-Chol-ICG-LP exhibited higher tumor accumulation.

**FIGURE 6:**
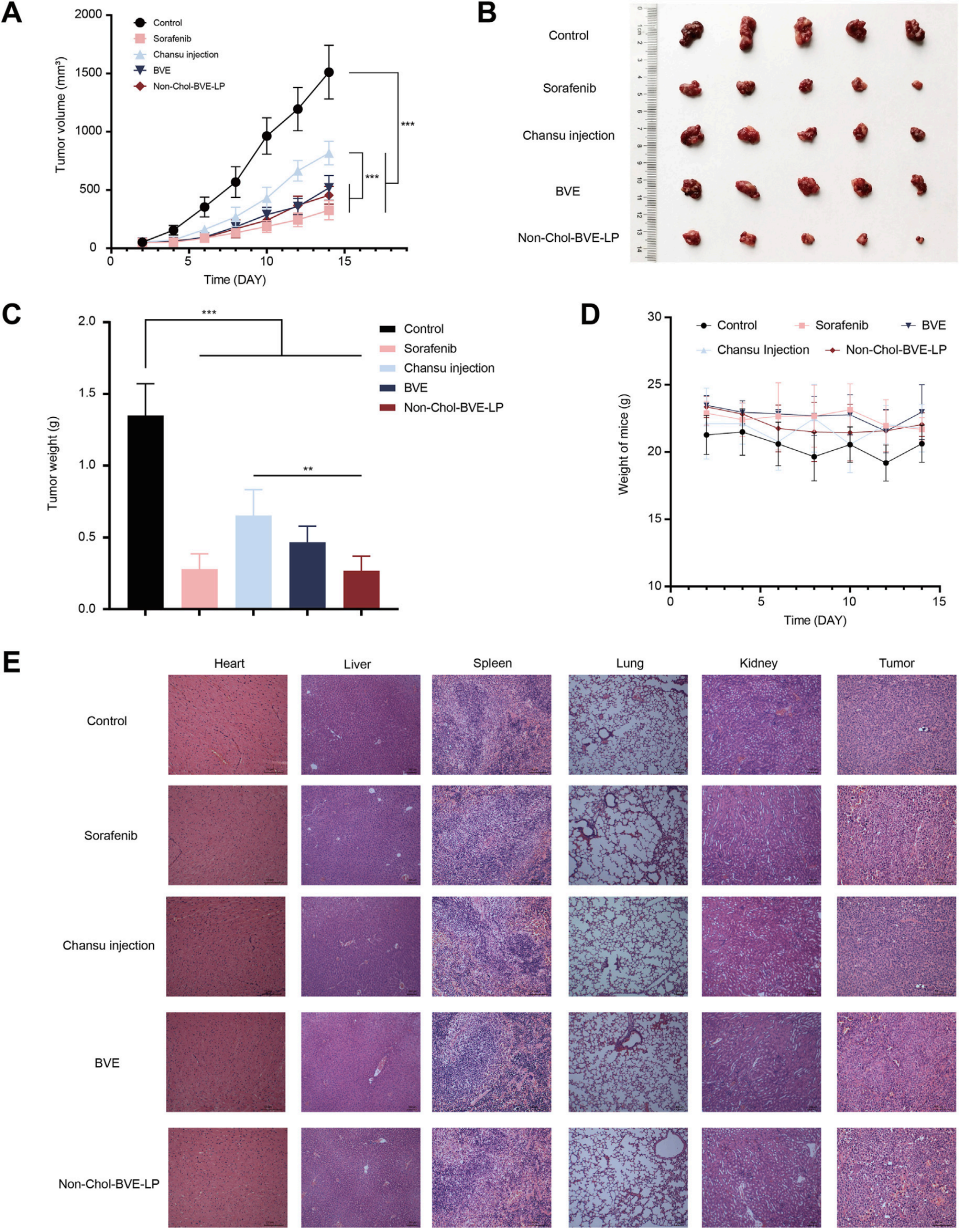
**(A)** Tumor growth curves of each group of tumors **(B)** Photographs of each group of tumors **(C)** Weight of each group of tumors **(D)** Body weight change curves **(E)** Histopathology sections of mice treated with various groups. Data is expressed as mean ± SD (n = 5, *P < 0.05, **P < 0.01, ***P < 0.001).

The anti-tumor effect on HepG2 subcutaneous tumor mice was investigated. As it was shown (Figures 6A–C), the tumor volumes in control, sorafenib, Chansu injection, BVE and Non-Chol-BVE-LP groups were 1511.02 ± 230.39 mm^3^, 326.34 ± 81.82 mm^3^, 816.84 ± 100.70 mm^3^, 577.72 ± 106.90 mm^3^ and 454.93 ± 76.43 mm^3^. The tumor weights were 1.34 ± 0.22 g, 0.28 ± 0.11 g, 0.65 ± 0.18 g, 0.46 ± 0.11 g and 0.26 ± 0.10 g respectively. And the tumor inhibition rates in sorafenib, Chansu injection, BVE and Non-Chol-BVE-LP groups were 79.75% ± 4.98%, 52.20% ± 6.70%, 65.75% ± 3.75% and 80.42% ± 5.69%. The above results indicated that liposomes had significant tumor suppressor effects with equivalent anti-tumor efficacy to the first-line anti-HCC drug sorafenib. Moreover, no significant changes in body weight of mice after treatment (Figure 6D). H&E staining results showed significant apoptosis and necrosis in the tumor tissue of the sorafenib and Non-Chol-BVE-LP groups, with typical features such as cell shrinkage and deepening of nuclear staining (Figure 6E). Therefore, Non-Chol-BVE-LP has achieved efficient anti-tumor effects by loading bufadienolides and indolealkylamines.

## 4 Conclusion

The presented studies provided a strong evidence of the likelihood of preparing cholesterol-free liposomes by replacing cholesterol with anti-tumor steroid such as bufadienolides. The liposome showed good size distribution, encapsulation efficiency drug loading capacity and slower drug release. Compared to BVE, Non-Chol-BVE-LP had higher cytotoxicity on HepG2 cells and induced more apoptosis on HepG2 cell. In addition, the liposomes had better intracellular uptake by HepG2 cells than the free drug, enhancing the drug accumulation in tumor, which facilitated the anti-tumor effects. *In vivo*, Non-Chol-BVE-LP showed high tumor accumulation and equivalent anti-tumor efficacy to the first-line anti-HCC drug sorafenib. The study provided new ideas for the development and clinical application of *Bufonis venenum* related formulation and offered new drug for the treatment of HCC.

## Data Availability

The original contributions presented in the study are included in the article/Supplementary Material, further inquiries can be directed to the corresponding authors.
