# Supplementary material for: Bufonis venenum extract loaded novel cholesterol-free liposome for the treatment of hepatocellular carcinoma
Source: Front Pharmacol. 2024 Nov 25;15:1486742. doi: 10.3389/fphar.2024.1486742 (PMC11625546; doi:10.3389/fphar.2024.1486742)
Supplement: Supplementary file 1 [file DataSheet1.docx]

***Bufonis venenum* extract loaded novel cholesterol-free liposome for the treatment of hepatocellular carcinoma**

Siqi Yang^1†^, Jinshuai Lan^1,2†^, Zhe Li^1,2^, Ming Li^1,^, Ya Wu^1^, Liyan Sun^1^, Tong Zhang^1,2 *^, Yue Ding^1,2,3 *^

^1^ School of Pharmacy, Shanghai University of Traditional Chinese Medicine, Shanghai 201203, China;

^2^ State Key Laboratory of Integration and Innovation of Classic Formula and Modern Chinese Medicine, Shanghai University of Traditional Chinese Medicine, Shanghai 201203, China;

^3^ National Innovation Platform for medical industry-education integration, Shanghai University of Traditional Chinese Medicine, Shanghai 201203, China；

^†^These authors have contributed equally to this work.

***** Corresponding authors.

Email address: zhangtongshutcm@hotmail.com (T. Zhang), dingyue-2001@hotmail.com (Y. Ding)

**Figure capture**

**Table. S1 The content of main components of BVE**

**Figure S1.** Particle size and Zeta potential images of different prescriptions liposomes

**Figure S2.** Changes in particle size of different prescriptions liposomes during 30 days

**Figure. S3** The stability of Non-Chol-BVE-LP in saline and MEM

**Figure. S4** Cytotoxicity of BVE and Non-Chol-BVE-LP on LO2 cells

**Table. S1 The content of main components of BVE**

| **Component** | **Content** |
| --- | --- |
| Gamabufotalin | 11.07 ± 0.05 mg/g |
| Arenobufagin | 17.96 ± 0.47 mg/g |
| Bufotalidin | 7.2 ± 0.09 mg/g |
| Deacetylcinobufotalin | 0.91 ± 0.11 mg/g |
| Bufotaline | 19.68 ± 0.61 mg/g |
| Cinobufotalin | 27.81 ± 1.30 mg/g |
| Telocinobufagin | 11.30 ± 0.36 mg/g |
| Deacetylcinobufagin | 2.09 ± 0.13 mg/g |
| Bufalin | 28.70 ± 1.08 mg/g |
| Resibufogenin | 39.40 ± 1.64 mg/g |
| Cinobufagin | 58.34 ± 2.65 mg/g |
| 5-Hydroxytryptamine | 0.66 ± 0.07 mg/g |
| Bufotenidine | 4.65 ± 0.89 mg/g |
| Bufotenine | 0.13 ± 0.04 mg/g |


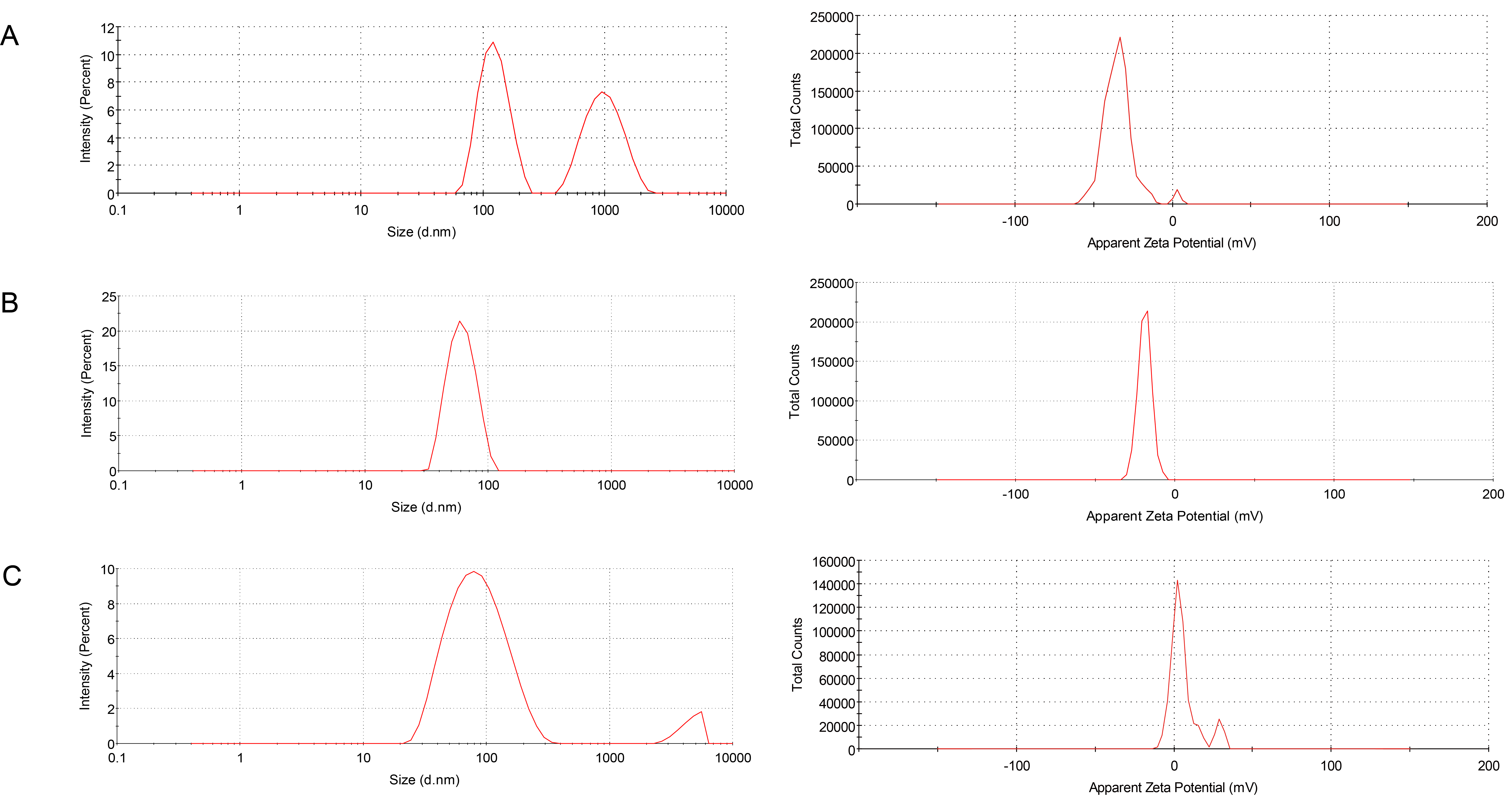


**Figure. S1** Particle size and zeta potential of Non-Chol-BVE-LP A) DSPC : DSPE-mPEG2000 : BVE =28.16 : 25 : 5.90 B) DSPC : DSPE-mPEG2000 : BVE =28.16 : 25 : 4.28 C) DSPC : DSPE-mPEG2000 : BVE =28.16 : 25 : 2.80


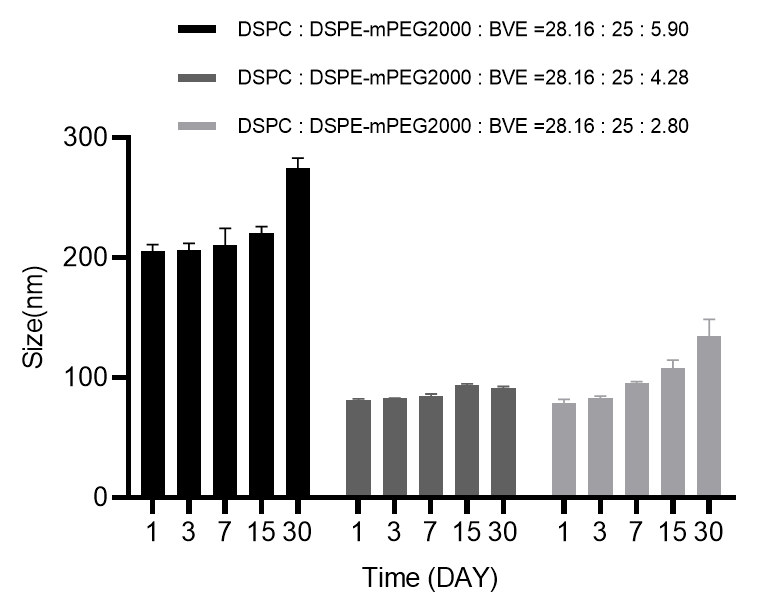


**Figure. S2** Changes in particle size of different prescriptions liposomes during 30 days (n=3)


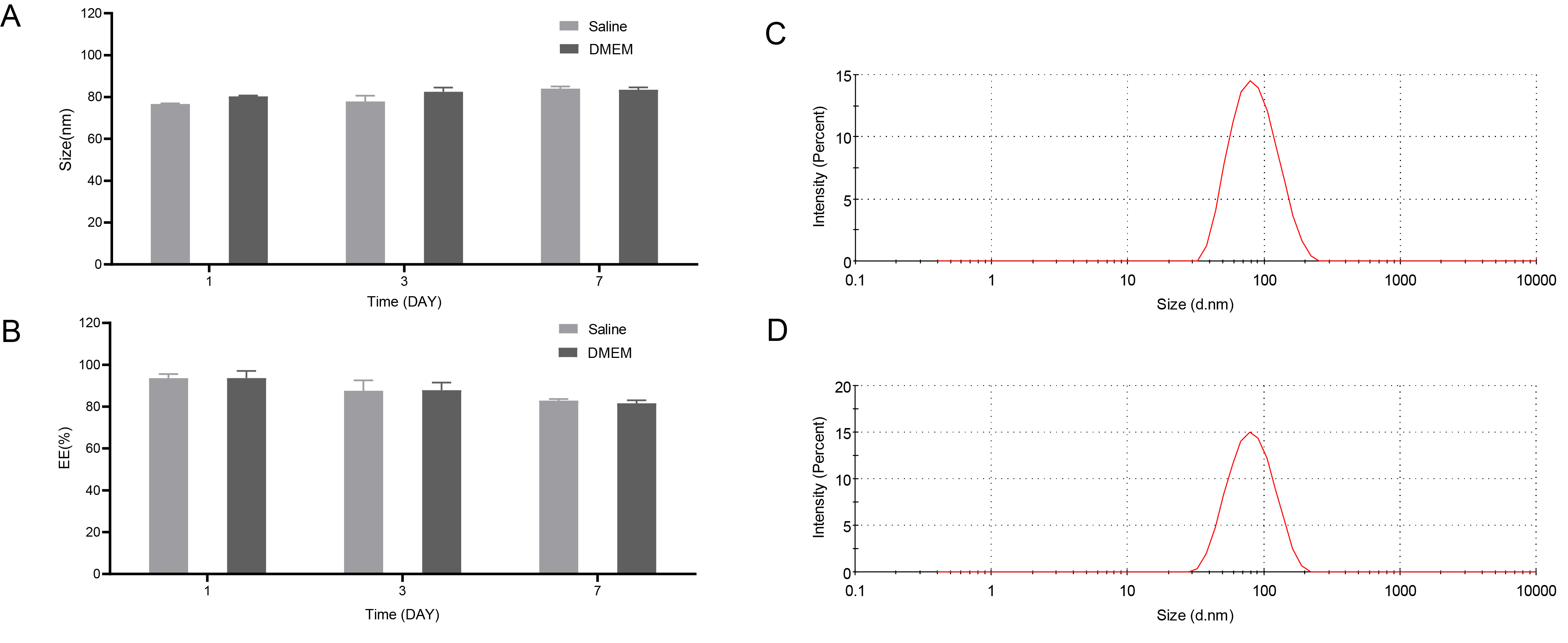


**Figure. S3** A) Changes in particle size of Non-Chol-BVE-LP in saline and MEM. (n=3) B) Changes in EE of Non-Chol-BVE-LP in saline and MEM. (n=3) C) Particle size of Non-Chol-BVE-LP in saline. D) Particle size of Non-Chol-BVE-LP in MEM.


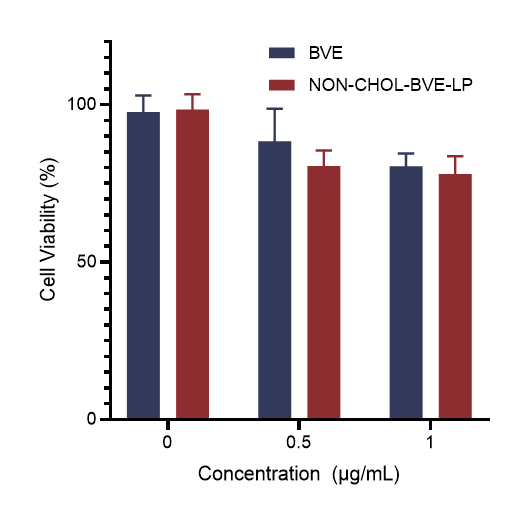


**Figure. S4** Cytotoxicity of BVE and Non-Chol-BVE-LP on LO2 cells
